#  Preparation and *in-vitro* Evaluation of an Antisense-containing Cationic Liposome against Non-small Cell Lung Cancer: a Comparative Preparation Study 

**Published:** 2013

**Authors:** Mostafa Saffari, Farshad H. Shirazi, Mohammad Ali Oghabian, Hamid Reza Moghimi

**Affiliations:** a *Department of Pharmaceutics, School of Pharmacy, Shahid Beheshti University of Medical Sciences, Tehran, Iran. *; b *Research Center for Science and Technology in Medicine, Tehran University of Medical Sciences, Tehran, Iran.*; c *Department of Pharmacology and Toxicology, School of Pharmacy, Shahid Beheshti University of Medical, Pharmaceutical Research Center, P.O.Box 14155-3817, Tehran, Iran. *; d *Pharmaceutical Sciences Research Center, Shahid Beheshti University of Medical Sciences, Tehran, Iran.*

**Keywords:** Gene delivery, Liposome, Thin film hydration, Ethanol injection, Cytotoxicity

## Abstract

The current methods for treatment of cancers are inadequate and more specific methods such as gene therapy are in progress. Among different vehicles, cationic liposomes are frequently used for delivery of genetic material. This investigation aims to prepare and optimize DOTAP cationic liposomes containing an antisense oligonuclotide (AsODN) against protein kinase C alpha in non-small cells lung cancer (NSCLC).

To perform this investigation, two different methods of ethanol injection and thin film hydration were used to prepare AsODN-loaded DOTAP liposomes.

The formulated liposomes were then evaluated for their morphology, particle size, zeta potential and encapsulation efficiency, and the best formulation was chosen. *In-vitro *growth inhibitory effect of encapsulated ODN on A549 cells were evaluated by MTT and colonogenic assay. The physical and serum stability of liposomal ODN were also evaluated.

Thin film hydration method resulted in large liposomes that required downsizing by extrusion with an encapsulation efficiency of 13%. Ethanol injection, in a single step gave liposomes with a small size of 115 nm and an encapsulation efficiency of around 90% which were physically stable for 6 months. The optimized liposome could protect oligonucleotides from degradation by nuclease. Cell studies showed a 20% sequence-specific inhibition of cell growth in MTT assay and revealed an LC_50 _of 103 nM in colonogenic studies.

In conclusion, ethanol injection was able to provide suitable liposomes from the permanently charged DOTAP. Also the resulted liposomes were able to inhibit the growth of lung cancer cells.

## Introduction

New therapies have been under investigation for development of strategies that target specific cellular proteins associated with tumor growth (1). Among these, antisense oligonucleotides (AsODN) which target the gene expression have seen the most exciting advances in this field (2). They all rely on the ability of oligonucleotide to interact directly with a specific intracellular target (3). However, the stability and delivery of these agents are yet to be optimized for a successful application (4, 5). Nuclease degradation before activity and the inability to penetrate cellular membranes are the major drawbacks of antisense oligodeoxynucleotides (6). Therefore, using suitable carriers is absolutely necessary for their successful therapeutic activity. 

Liposomes are one of the most extensively used carriers in gene delivery (7). Oligonucleotides have negative charge and among different type of liposomes, cationic vesicles have been shown to provide higher encapsulation efficiency for them due to the electrostatic interactions. Cationic liposomes are prepared by using cationic lipids like di-octadecenyl-trimethylammonium propane (DOTMA), dioctadecyldimethylammoniumbromide (DDAB), dioctadecylglycerylspermine (DOGS), dioleoyl dimethylaminopropane (DODAP), and bis (oleoyloxy)-3-(trimethylammonio) propane (DOTAP). 

Among different methods, thin film hydration is the most widely used method for preparation of cationic liposomes (8, 9); although, most of the reported results from this method showed inappropriate encapsulation efficiency (EE), size, stability and homogeneity of liposomes. Semple et al. showed that ethanol injection could provide high encapsulation efficiency (up to 70%) of hICAM antisense using DODAP as the cationic lipid for liposomes. Their method used the pH of 4 at the beginning at which DODAP is positively charged, followed by another pH (pH = 7.4) at which DODAP is neutral (10). 

On the other hand, among different cationic lipids some lipids that are widely used in preparation of cationic liposomes like the DOTAP (11, 12), are permanently positively charged, regardless of the pH (13). This investigation aimed to prepare DOTAP cationic liposomes containing AP1261 as an ODN against protein kinase C-alpha (PKC-*α*). This cationic lipid is a frequently used lipid in preparation of liposomal gene delivery systems, but opposite to DODAP, it is permanently charged. As there is no report on application of ethanol injection method for DOTAP, this investigation also aimed to study the application of this method for preparation of DOTAP containing liposomes and compare the resulted liposomes with those prepared by thin film hydration. Besides, the effect of sonication on the ethanol injection method has also been investigated here. 

After comparing methods, the best formulation was chosen and used for cell culture studies. One potential target of cancer gene therapy is PKC-*α*, a signaling molecule with an important role in cell regulation and proliferation (14). This study used AP1261 as an AsODN which has been shown to specifically down-regulates PKC-*α *in A549 cells as a non-small cell lung carcinoma (NSCLC) (15). 

## Experimental

Distearoylphosphocholine (DSPC) was purchased from Northern Lipids (Vancouver, Canada). PEG2000-DSPE and Egg phosphocholine were purchased from Lipoid (Germany). DOTAP, Cholesterol (Chol), Bromophenol blue, Polycarbonate filters, Sepharose DEAE, Triton X-100, 1,8 Cineole, Limonene, TEMED and ethidium bromide were obtained from Sigma-Aldrich Chemical Company (St. Louis, USA). A 20-mer phosphorothioate modified AsODN 5’-TsCsCs AsTsGsAsCsGsAsAsGsTsAsCsAsGsCs CsGs-3’ directed against protein kinase C-*α *mRNA -synthesized by Bioneer (korea)- was used as a previously validated model of antisense therapy in non-small cell lung cancer (15). Agarose were purchased from Merck (Germany). Fetal bovine serum was from Gibco BRL. All other reagents were of analytical grade.

*Preparation of liposomes by thin film hydration*

Different formulations of ODN-containing liposomes were prepared. Neutral liposomes composed of Chol/DSPC (45:55) were prepared by thin film hydration method (TFH) as described previously (16). Briefly, lipid components of the liposomes were dissolved in chloroform/methanol and heated in a rotary evaporator to remove the organic phase. Afterwards, ODNs were dissolved in HEPES buffer saline (HBS, pH = 7.4) and the lipid layer was hydrated by this solution to form MLV liposomes. The suspension was then extruded 3 times through 100 nm polycarbonate membrane for size reduction.

As the next step, DOTAP as the cationic lipid along with PEG2000-DSPE were added to the above mentioned formulation and the cationic liposomes containing DOTAP:DSPC:Chol:PEG2000-DSPE were prepared by TFH as described above. This system used the ratios of 25:20:45:10 (respectively) as previously used for DODAP and PEG-ceramide containing liposomes (10). 

*Preparation of liposomes by ethanol injection method*

Ethanol injection method was used as described by Semple and his coworkers (10). For preparation of liposomes by this method, the above-mentioned lipid mixture was dissolved in absolute ethanol and this cocktail was injected gently under vigorous shaking into ODN solution in citrate buffer (pH = 4). To remove ethanol, liposomes were dialyzed for 1 hour in citrate buffer (pH = 4), followed by dialysis in HEPES buffered saline (HBS, pH = 7.5) as described by Semple *et al. *(10). Residual free ODNs were subsequently removed using Sepharose DEAE and gel filtration chromatography through a previously conditioned column with HBS (pH = 7.5). 

Finally, to evaluate the potential influence of sonication on properties of the ethanol-injection liposomes, two sonication cycles of 5 minutes each were performed on liposomes at their initial stage before dialysis.

*Particle size and zeta-potential determination*

Zeta potential and particle size of liposomes were determined by Malvern Zetasizer (UK). Liposomes were diluted with HBS, and measurements were carried out at 25°C under specified conditions (viscosity=0.88 cP and reflex index=1.33).

*Determination of ODN encapsulation efficiency*

ODN encapsulation efficiency is expressed here as the recovered ODN/lipid weight ratio compared to the initial quantities in each formulation. The recovery of ODN and lipid were determined separately as follows: ODN content was determined by UV-spectrophotometery (Shimadzu, Japan) at 260 nm after liposome solubilization in chloroform/methanol (1:2.1). Standard solutions of ODN in concentration range of 0-50 μg/mL were used for obtaining the standard curve. Linear regression was performed and showed that the assay method was valid for ODN concentrations in the applied range. Phospholipid content was determined after lipid extraction by Stewart method which is a method based on formation of a complex between phospholipids and ammonium ferrothiocyanate (17).

*Transmission electron microscopy*

ODN-containing Liposomes were mixed with 1% sodium phosphotungstate (pH 7.0) for a few seconds and were pipetted onto coated copper grids. After removal of the excess stain by filter paper, the samples were viewed and photographed with an LEO transmission electron microscope (Germany) at an accelerating voltage of 80 KV (18).

*Physical stability*

Liposomes were kept at 4°C for 6 months and were analyzed according to their size and zeta potential at time zero, and after 3 and 6 months.

*Serum stability of ODN*

The biological stability of liposomal ODN was compared with free ODN. Liposomal or free ODN were incubated with fetal bovine serum at 37°C for 6 hours. Serum enzymes were inactivated by heating the final mixture at 70°C for 15 min. In addition, samples of liposomal ODN were treated with Triton X-100 (to disrupt the liposomes and release their ODN contents) either without any exposure to serum or after such exposure, as the test and negative control respectively. After the exposure period, the intact or degraded ODN were assessed by gel electrophoresis as described below.

Gel electrophoresis was performed on 2% agarose gel, containing ethidium bromide for visualization, at 70 V for 30 minutes (19). The intensities of the resulted bands in the captured gel image were analyzed with G-Box instrument and software (Silk Scientific Corporation).* Statistical analysis*

Comparison of the percent viability, survival fractions and liposomes’ characteristics among the different groups were performed using one-way analysis of variance (ANOVA) and Tukey or bonferroni post-hoc. Standard curves were generated based on linear regression and the prerequisite for statistical significance was considered to be as p < 0.05.

*Cell culture studies*

A549 cells, obtained from Pasteur Institute (Tehran, Iran), were grown in RPMI 1640 medium supplemented with 10% heat-inactivated FBS, 100 U/mL penicillin and 100 μg/mL streptomycin. They were maintained at 37^◦^C in a 5% CO_2_-incubator (Heraeus, Germany). Cell viabilities were evaluated by cell count using trypan blue stain 0.4% under light-inverted microscope (Leica, Germany) and the cytotoxicities of the optimized liposomes were evaluated by MTT (20) and colonogenic assay.

*MTT assay*

10000 of A549 cells were seeded in each well of 96-well plate and were incubated at 37^◦^C in 5% CO^2^ for 24 h. Afterwards, the cells were treated with different liposomal ODN concentrations (AsODN or ScODN) for 48 h. Then, 20 μL of MTT (5 mg/mL) was added to each well and incubated for 4 hours in incubator. The medium containing MTT was then aspirated and 100 μL of dimethyl sulphoxide (DMSO) was added to each well, and the plates gently agitated until the formed formazans were dissolved. The absorbance of each well was read using a plate reader (Rainbow, Australia) at 570 nm, subtracting the absorbance at 650 nm as a reference (there is no absorbance by MTT at 570 nm).

*Colonogenic assay*

A549 cells were cultured as a monolayer in RPMI 1640 culture media. After 80 % confluence, the attached cells were harvested by trypsin treatment. Then an aliquot of the sample was taken and counted under inverted light microscope using a hemocytometer to determine the cell density. The sample was then diluted to 50000 cells/mL. After 24 h, the cells were treated by liposomal ODN (AsODN or ScODN) for 48 hours after which they were rinsed by normal saline and the monolayer were trypsinized and seeded in 6 well plates for 10 days. The cells were then fixed, stained and allowed to dry. The colonies were then counted, considering the limit of 50 cells for each colony (21) and the results were compared with control (3 wells for each), based on surviving fraction. Different groups were compared by ANOVA with Bonfferoni correction and non-linear regression was applied to estimate IC_50_ of the formulated AsODNs-containing liposome.

## Results and Discussion

*Characterization of liposomes and comparison of the preparation methods*

Neutral liposomes showed an encapsulation efficiency of 8.2 ± 1.4 % (n = 3) which is considered low, but is in agreement with the previously published data of other neutral liposomes by TFH method (22, 23). These liposomes showed two populations of 120 nm and 1 μm (Table 1).

**Table 1:** Characteristics of the different ODN-containing liposomes (n = 3).

*%**EE**	**Preparation Method**	**Type**	**Size**	**.Formulation No**
1.4 ± 8.2	TFH ٭*	Neutral	nm and 1μm 120	1
1.08 ± 13	TFH	Cationic	μm 125	2
3.8 ± 87.5	Ethanol injection	Cationic	nm 115	3
3.2 ± 83.2	Ethanol injection + Sonication	Cationic	nm 164	4

*Encapsulation Efficiency. **Thin Film Hydration

Size and Zeta-potential of cationic liposomes are shown in Table 1 and as is observed, passive ODN encapsulation in cationic liposomes has slightly increased the encapsulation efficiency to about 13% and has made the distribution of particles more uniform (Table 1).

Ethanol injection method resulted in vesicles with a size of 115 nm not requiring downsizing with extrusion or other methods. Besides, this system provided an encapsulation efficiency of around 90% that was about 7 times more than that of the thin film hydration method. This level of EE is in agreement with that of DODAP-containing liposomes (70%) reported by Semple *et al. *(10). This huge difference between the TFH and ethanol injection methods could be due to the lower possibility of interaction of cationic lipids and anionic ODNs in TFH due to lower contact surfaces. Apparently, this effect cannot be overcome by subsequent downsizing methods as extrusion. As described by Semple *et al.*, ethanol injection method facilitates the interaction of lipids and oligonucleotide due to destabilization of liposomal membranes and finally makes vesicles in which ODNs are sandwiched between bilayers (10).

The present study used DOTAP and the results showed higher encapsulation efficiency than that reported by Semple *et al. *(10) and Tamaddon *et al. *(24) for DODAP-containing liposomes. Lower EE in DODAP containing liposomes might be due to ODN loss after neutralization of DODAP at pH of 7.4. However, this conclusion might require further experiments for confirmation. 

The present system had a zeta potential of 0.6 ± 0.7 mV (mean ± SD, n = 3) which did not differ from zero significantly (p > 0.05). As DOTAP is permanently charged, this neutral zeta potential that is close to what reported for DODAP containing liposomes (24) might support the above-mentioned discussion regarding higher EE. Application of sonication was not able to improve the properties of liposomes (regarding the size and EE); instead, the size was slightly increased and EE slightly decreased (Table 1). Among the above mentioned formulations, the third formulation (F_3_, Table 1) was selected as the best liposomal system and extra characterizations were implemented on this formulation. This system provided particles with suitable mean diameter and polydispersity index (0.14) in a single step without extrusion.

*Transmission electron microscopy*

**Figure 1:**
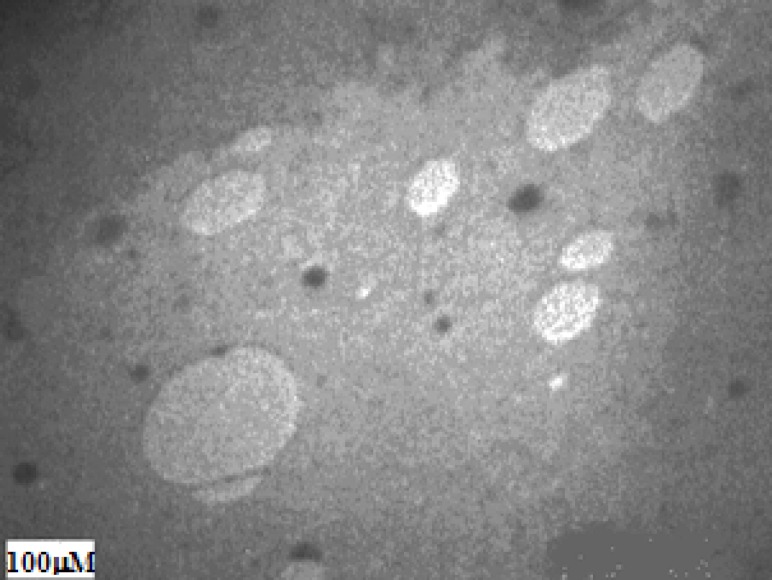
Electron microscopic image of the antisense oligodeoxynucleotide-encapsulating liposome (F_3_).

The liposomes were negatively stained and photographed by an electron microscope to estimate their size distribution and characterize the morphology (Figure 1). 

These liposomes seem to be pear like and small. Their complex morphology is in accordance with the SALPs prepared by DODAP (10), and consists of a mixed population of unilamellar and small multilamellar vesicles (around 100 nm in diameter).

*Physical stability*

**Figure 2:**
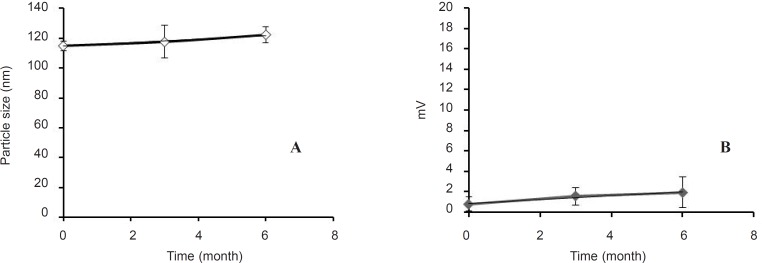
Stability of the optimized antisense oligodeoxynucleotide-encapsulating liposomes (F3) during 6 months as evaluated by their size (a) and zeta potential (b), (n = 3).

Figure 2 provides the size and zeta potential of cationic liposomes prepared by ethanol injection after 6 months storage at 4°C. The results show that there was no change in their properties (analyzed by ANOVA between different time points). Liposomes were stable in refrigerator (4°C) for this period of time and did not change significantly (p > 0.05).

*Stability of liposomal ODNs against enzymatic degradation in serum*

**Figure 3:**
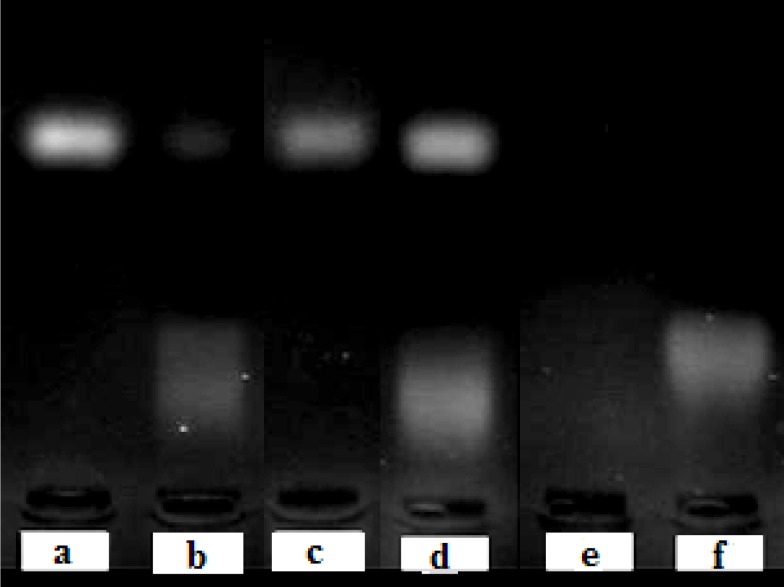
Stability of free ODN in absence (a) or presence (b) of fetal bovine serum and the liposomal ODN in absence (c) or presence (d) of fetal bovine serum at 37°C for 6 h. Line (e) is pure equivalent total lipids and line (f) shows pure serum as control. Stability was analyzed by 2 % agarose gel electrophoresis, 70V for 30 min.

Stability studies showed that free ODN was degraded completely in presence of serum, but liposomal encapsulation of ODNs protected them from degradation (Figure 3). 

Liposomal ODNs and free ODNs without exposure to FBS were used as control both showing clear band for intact ODNs (Figure 3). After exposure of liposomes to serum and inactivation of serum enzymes by heat, cationic liposomes were broken by Triton X-100 to release their ODN content (Figure 3, d). They showed clear band which implies full protection of ODN from nuclease degradation.

The results indicated that the electrostatic binding and encapsulation of ODNs in cationic liposome was sufficient to provide protection against nuclease degradation as was previously reported for some other gene delivery carries (19, 10).

*Colonogenic assay for evaluation of A549 cell growth inhibition effect*

**Figure 4:**
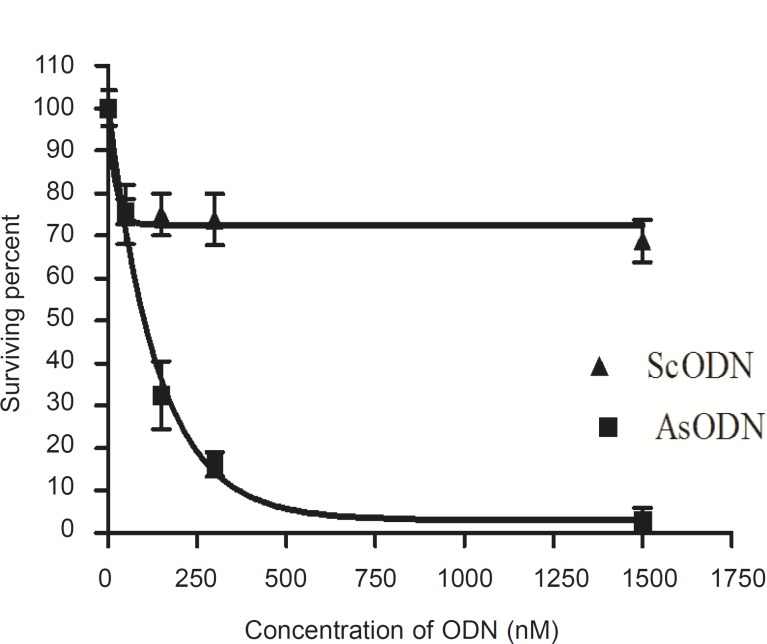
Colonogenic growth inhibitory effect of liposomal antisense oligodeoxynucleotide (AsODN) upon 48 h incubation with A549 cells compared to scrambled oligodeoxynucleotides (ScODN, n = 3).

Liposomal AsODN showed a significant dose-dependent antiproliferative activity (Figure 4, p < 0.05). Exponential linear model was fitted to curve and the IC_50_ of liposomal AsODNs after 2-days incubation of A549 cells with liposomal AsODN was calculated as about 103 nM. This IC_50_ was comparable with the results of Song and co-workers who reported an IC_50 _of less than 100 nM in their work, although they applied lipofectin which had excess positive charge (15). Besides, the IC_50_ was statistically lower than the IC50 of ISIS 3521 that is also a 20 mer ODN which acts against PKC-*α *(25).

Comparison of the survival percent for AsODN and ScODN (Figure 4) indicates a sequencespecific inhibitory effect of our antisense ODNencapsulating cationic liposomes.

*Inhibition of cell proliferation by MTT assay*

**Figure 5:**
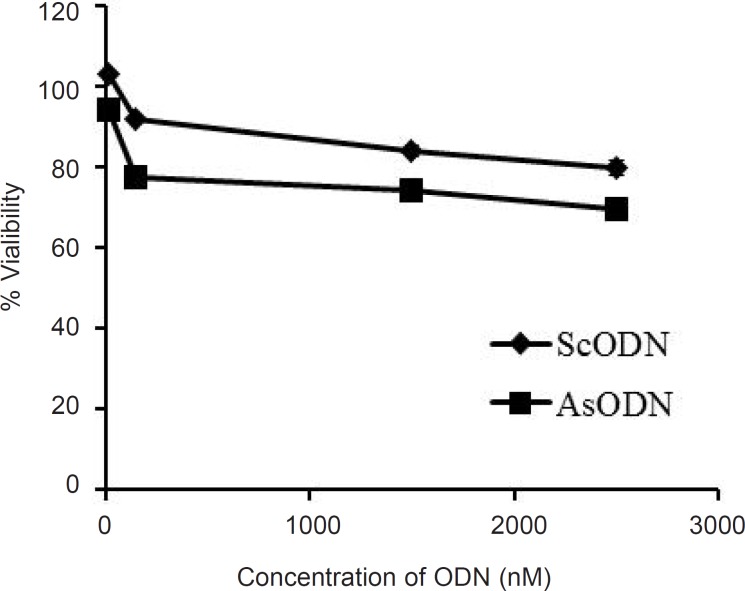
Antiproliferative effect of the liposomal antisense oligonucleotide (AsODN) in comparison to its control (ScODN) at different concentrations after 48 h exposure time. Data are mean ± standard error (n = 6).

The antiproliferative ability of liposomal antisense ODN at a concentration range of 15 to 2500 nM in culture medium was investigated on A549 cells and compared with that of the ScODN-containing liposomes. The inhibitory effect of ODNs on cell proliferation was determined after 48 h of transfection using the MTT assay. As shown in Figure 5, ScODNs had almost no significant effect on proliferation of A549 cells. However, liposomal AsODNs significantly reduced cell viability and brought about an inhibition activity of 20% in A549 cells. At 150 nM of ODN, the difference between AsODN and ScODN (which is defined as a sequence-specific inhibitory effect) was at maximum and after this point it seems that the non-specific toxicity of ScODN is increased. The increase in non-specific cytotoxicity after an optimum point has also previously been reported by Tamaddon (24).

## Conclusion

The present data show that ethanol injection is a good method for preparation of DOTAPcontaining cationic liposomes and can provide small particles with high encapsulation efficiency in a single step. The traditional film hydration technique resulted in large liposomes that required using further downsizing techniques as extrusion. Besides, film hydration method resulted in very low encapsulation efficiency. The prepared liposomes showed sequence-specific cell growth inhibition and might be used for *in-vivo *evaluation.
